# In-Service Detection and Quantification of Railway Wheel Flat by the Reflective Optical Position Sensor

**DOI:** 10.3390/s20174969

**Published:** 2020-09-02

**Authors:** Run Gao, Qixin He, Qibo Feng, Jianying Cui

**Affiliations:** 1 Key Lab of Luminescence and Optical Information, Ministry of Education, Beijing Jiaotong University, Beijing 100044, China; 17118454@bjtu.edu.cn (R.G.); qbfeng@bjtu.edu.cn (Q.F.); jycui1@bjtu.edu.cn (J.C.); 2 Dongguan Nannar Electronic Technology Co., Ltd., Dongguan 523050, China

**Keywords:** railway wheel, condition monitoring, laser collimation, wayside measurement

## Abstract

Railway wheel tread flat is one of the main faults of railway wheels, which brings great harm to the safety of vehicle operation. In order to detect wheel flats dynamically and quantitatively when trains are running at high speed, a new wheel flat detection system based on the self-developed reflective optical position sensor is demonstrated in this paper. In this system, two sensors were mounted along each rail to measure the wheel-rail impact force of the entire circumference by detecting the displacement of the collimated laser spot. In order to establish a quantitative relationship between the sensor signal and the wheel flat length, a vehicle-track coupling dynamics analysis model was developed using the finite element method and multi-body dynamics method. The effects of train speed, load, wheel flat lengths, as well as the impact positions on impact forces were simulated and evaluated, and the measured data can be normalized according to the simulation results. The system was assessed through simulation and laboratory investigation, and real field tests were conducted to certify its validity and correctness. The system can determine the position of the flat wheel and can realize the quantification of the detected wheel flat, which has extensive application prospects.

## 1. Introduction

With the development of railway transportation towards faster speeds and larger axle loads, the wheel-rail contract force is increasing, which puts forward higher quality requirements on the wheels. Wheel flat is the most common local surface defect in the service life of railway wheels. The reason for this defect is mainly related to abnormal braking of the vehicle, and may also be caused by low wheel-rail adhesion due to environmental conditions (rain, snow, leaves, etc.), which makes the wheel slide locally on the rail surface [1,2,3]. Wheel flats will bring additional periodic impact load to the rail during vehicle operation, thus giving rise to high impact forces in the wheel-rail interface. The deterioration is the inevitable result of increasing wheel-rail contact force. Uncontrolled deterioration can, however, lead to wheel bearing damage, axle temperature increase, axle fracture, rail and concrete sleeper fracture, and rail deformation aggravation, which will seriously affect train safety [4,5,6,7]. The rapid and accurate detection of overrun flat wheels has become the most important issue for researchers.

At present, the commonly used in-service detection methods of wheel tread flats mainly include ultrasonic methods [3,8,9], the parallelogram method [10,11], and the impact load detection method [12,13,14,15,16,17,18,19,20,21,22,23]. Ultrasonic methods are being intensely used for non-destructive evaluation, which can be divided into the electromagnetic ultrasonic method and the ultrasonic ranging method according to different measuring principles. The electromagnetic ultrasonic method [8] is a contact detection method that assess the existence of flats directly with an electromagnetic ultrasound sensor as a probe, which is simple in structure and low in cost. The ultrasonic ranging method [3] is used to detect the wheel wear and flat length through the different round-trip time of Rayleigh ultrasonic pulse wave. The disadvantage is that the measurement accuracy is affected by the measurement distance, therefore it is difficult to accurately measure the flat on the entire circumference of the rolling wheel. The parallelogram method is another contact detection method based on the parallelogram mechanism [10]. If flats exist on the wheel tread, the position information of the measuring rule can be detected and the flat depth can be calculated by the amplitude of the signal [11]. Due to its own limitations, both the ultrasonic method and the parallelogram method are only suitable for low-speed measurement (the train’s speed is usually restricted to 15 km/h).

The impact load method is characterized by its wide application range, convenient installation, and low technical difficulty, and has been widely used in high-speed railway wheel flat detection. According to the to the installation location, the impact load method can be categorized as on-board and wayside measurements. The on-board method [14,15,16,17] installs the sensors, such as an accelerometer, on the axle box, and the acceleration signal can be used as the measurement signal. The wheel-rail contact state can be detected by analyzing the external excitation in the wheel-rail interaction, and then the wheel flat can be evaluated. The measurement results of the on-board method are easily affected by the factors such as rail gap, rail installation, bending, and so on, which will easily lead to misjudgment or missing judgment. The wayside method measures the wheel flats by installing a series of sensors on the rails and surrounding areas. At present, strain gauges [12,13], accelerometers [18,19], fiber optic sensors [20,21,22], and piezoelectric cable [23] are commonly used sensors to measure the wheel-rail impact forces. As the signal collected by these sensors is the wheel-rail coupling signal of three directions (lateral, longitudinal and vertical), the signal analysis and processing are difficult and complicated. Besides, the measurement results of strain gauge and accelerometer are susceptible to electromagnetic interference and the installation of these sensors will cause damage to the rails [20].

The diagnosis of wheel flats has been realized in the papers reviewed above, but its quantification remains a difficult point, due to the effects of the various measurement conditions. However, the quantification of wheel flats is essential to the prognosis for predicting future failures and the remaining useful life of wheels. In order to realize the detection of high-speed train wheel flats dynamically and quantitatively, an in-service detection system based on the self-developed reflective optical position sensor is demonstrated in this paper. The sensor has the advantages of great accuracy and sensitivity, fast response speed, electromagnetic immunity, and compact size. In addition, the sensors can be easily installed without causing damage to rails. In order to realize the quantitative measurement of the wheel flat length, the finite element simulation and the multi-body dynamics simulation were conducted to establish the vehicle-track coupling dynamics analysis model. The influence of various environmental situations and operational conditions such as speed, axle load, wheel flat lengths, as well as impact positions were analyzed. Then, the flat length was quantified according to the simulation results. Experiments were conducted in laboratory and railway site respectively and the correctness of the model simulation is verified based on the results of the experiments.

The rest of this paper consists of four parts. First in Section 2, the detection principle and system structure are described. Section 3 mainly treats the establishment of the vehicle-track coupling dynamic analysis model. In Section 4, the influence factors of wheel flats on rail deformation are analyzed, and wheel flat length is quantified. Section 5 describes the experiments carried out in the laboratory and in the field, respectively.

## 2. System Structure

The system consists of three modules: sensor module, data processing module, and data communication module. The sensor module was mounted along the rail and the data processing module and data communication module were installed in the monitoring room beside the track along with the data communication module. In the system, two reflective optical position sensors were mounted on each rail to detect the entire wheel tread circumference information, to avoid missing detection.

### 2.1. Sensor Design

**Figure 1:**
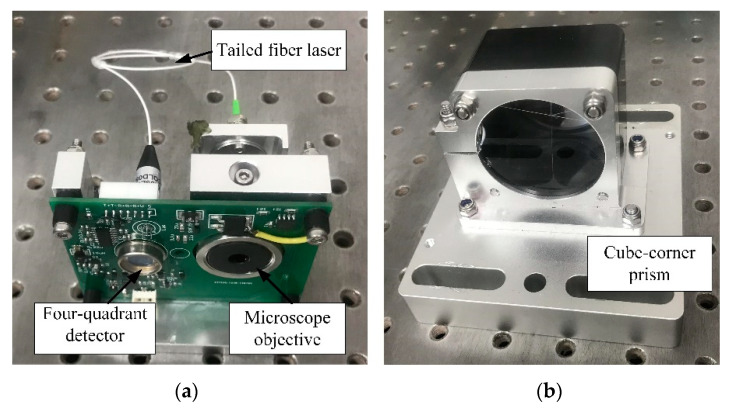
The picture of the sensor: (**a**) The light source and detection module; (**b**) The reflection module.

In the reflective optical position sensor, a tailed fiber laser and a four-quadrant detector were used to measure the rail deformation, as shown in Figure 1a. The tailed fiber laser and the four-quadrant detector were integrated by a self-developed integrated circuit board, which can realize functional modularization, and ensure that the laser operates synchronously with the detector. The peak wavelength of the tailed fiber laser (BWT Beijing LTD, model K635F03RN) is 632 nm, and the power is 2 mw. The laser beam was collimated by the microscope objective with a focal length of 9.25 mm. The light source and the detector were integrated and mounted on the rail by a clamp block (Part 1). A cube-corner prism was added to realize the reflection of the laser beam and to improve the sensitivity of the flat measurement. The cube-corner prism was integrated with two windows and two filters, which were mounted by another clamp block (Part 2), as shown in Figure 1b. The lateral and vertical rail bending deformation under the wheel-rail interaction can be acquired simultaneously by this sensor, so as to realize the separate measurement of wheel-rail coupling signal, which is conducive to the accurate extraction of flat signal.

### 2.2. Measurement Principle

**Figure 2:**
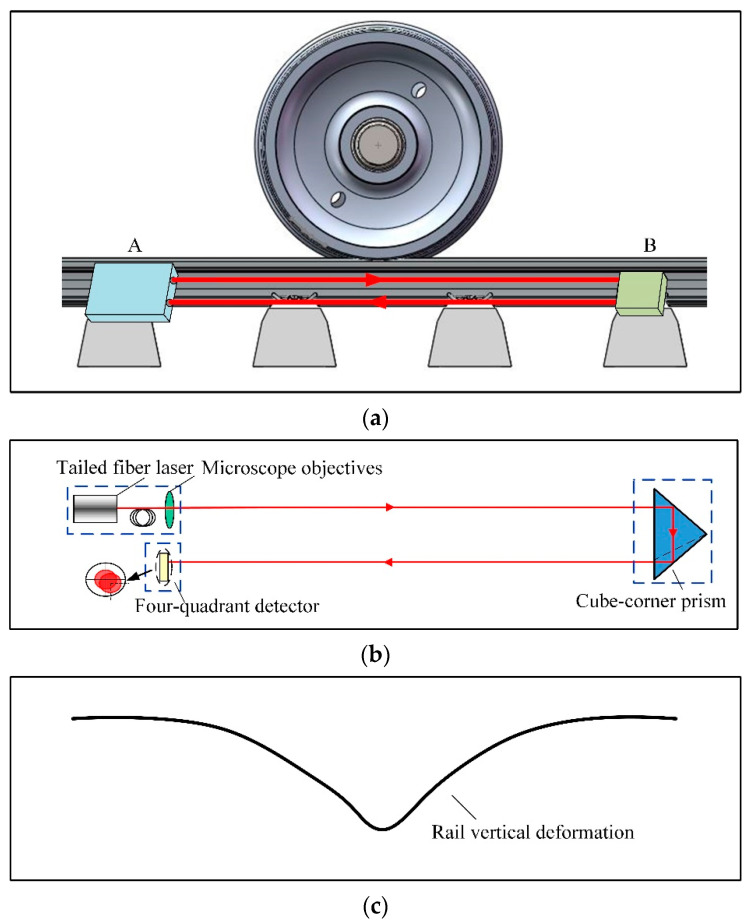
The schematic diagram of the installed sensor modules and the rail vertical deformation: (**a**) The installation location of the sensor; (**b**) The schematic diagram of the sensor; (**c**) The rail vertical deformation.

The collimated light with a spot diameter of 5 mm was generated after passing through the microscope objective, and then reflected into the four-quadrant detector with a detection diameter of 8 mm through the cube-corner prism. A schematic overview of the installed sensor modules and the rail vertical deformation is shown in Figure 2. In Figure 2a, Part 1 and Part 2 of the sensor were mounted on point A and B of the rail respectively to measure the rail deformation, and the schematic diagram of the sensors is shown in Figure 2b. In the initial state, the laser spot was located in the center of the four-quadrant detector’s target plane. When the wheels pass through, the rail deforms and the displacement of rail bending deformation is related to the magnitude of the wheel-rail contact force. Due to the rail deformation, a vertical displacement will occur on the sensor module mounted on the rail. At the same time, it will cause a variation on the angle of the sensor module, which also resulting in the change of the spot position in the four-quadrant detector. After the wheel passes through, the schematic diagram of the rail vertical deformation detected by the detector is shown in Figure 2c.

The continuous change of the spot position caused by different factors is analyzed as follows:

(1) The vertical displacement of Part 1

**Figure 3:**
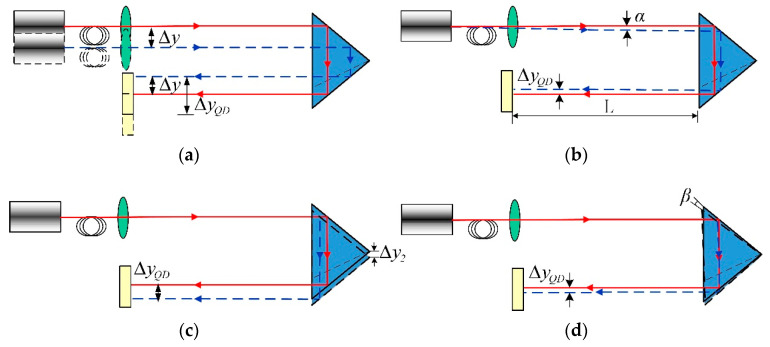
The optical path analysis diagram of laser collimation measurement system: (**a**) The schematic diagram of the vertical displacement of the sensor module; (**b**) The schematic diagram of the laser emission angle changes; (**c**) The schematic diagram of the vertical displacement of the reflection module; (**d**) The schematic diagram of the angle charge of the cube-corner prism.

As the laser, cube-corner objective, and four-quadrant detector were integrated together as Part 1, their positions changed synchronously under the wheel-rail contact forces. As shown in Figure 3a, the vertical displacement of Part 1 is Δy, the resulting deformation of the light spot on the detector can be represented as:

(1) $$\Delta y_{QD}\;=\;2\Delta y$$

(2) Laser emission angle

As the laser is fixed on the rail by a clamp, the laser emission angle will be affected by the rail deformation. As shown in Figure 3b, the laser angle change is α, based on the prism tunnel principle; the deformation value of the light spot on the detector can be expressed as:

(2) $$\Delta y_{QD}\;=\;2L\tan\alpha \;+\;\alpha \left(1\;-\;\frac{1}{n}\right)h$$

where *n* is the prism refractive index and *h* is the prism tunnel width.

(3) The vertical displacement of Part 2

The cube-corner prism, two windows, and two filters were integrated as Part 2. Under the wheel-rail contact forces, the vertical displacement of the cube-corner objective prism is $\Delta y_{2}$, as shown in Figure 3c. The deformation value of the light spot on the detector can be expressed as:

(3) $$\Delta y_{QD}\;=\;2\Delta y_{2}$$

(4) The angle of the cube-corner prism

As shown in Figure 3d, according to the prism tunnel principle, when the cube-corner prism rotates *β*, the deformation value of the light spot can be expressed as:

(4) $$\Delta y_{QD}\;=\;\beta \left(1\;-\;\frac{1}{n}\right)h$$

(5) Comprehensive analysis

Based on the above discussion, the deformation value of the light spot in the vertical position can be obtained as:

(5) $$\Delta y_{QD}\;=\;2\Delta y\;+\;2L\tan\alpha \;+\;\alpha \left(1\;-\;\frac{1}{n}\right)h\;-\;2\Delta y_{2}\;-\;\beta \left(1\;-\;\frac{1}{n}\right)h$$

The lateral position of the light spot in the detector is the same as the above analysis. If there is a flat on the wheel tread, an impact signal will occur in the deformation curve due to the additional wheel-rail impact force. By detecting the impact signal in the deformation curve, the wheel flat can be identified.

### 2.3. System Structure and Installation

**Figure 4:**
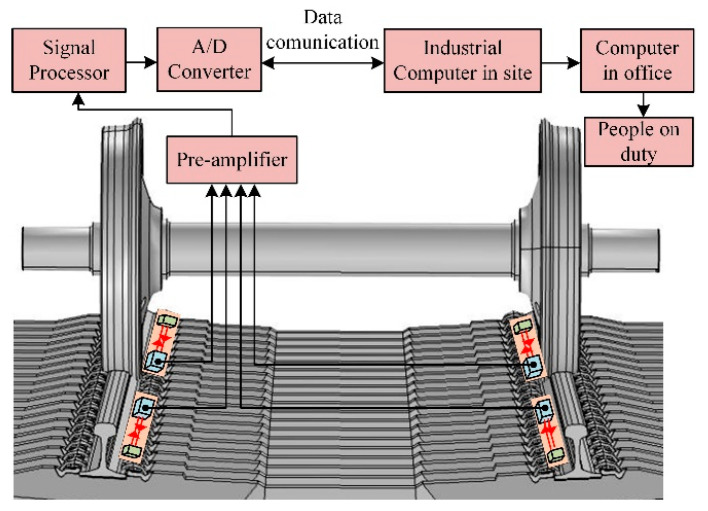
The schematic diagram of laser collimation measurement system.

The schematic view of the system is shown in Figure 4. In order to measure the flat on the entire circumference of the wheel, two reflective optical position sensors were mounted on each rail and the distances were calculated. The installation distance between the laser and the cube-corner prisms was set to 400 mm to realize a measuring range of 1.5 m. The distance between two sensors on each rail was 1.5 m, so the total measuring distance of the system was 3 m. When the train passed through the measurement system, the detection signal generated by the four-quadrant detector was fed to the computer through serial communication, and then processed by the computer. If the flat signal appeared in the measurement curve, the flat wheel could be located by analyzing the time sequence of the whole train waveform.

## 3. Vehicle-Track Dynamics Simulation Model

Multibody dynamics simulation has been widely used to analyze the complex motion relationships of objects. In order to further analyze the influencing factors of wheel flat detection, the vehicle-track coupling dynamic model was established based on the multi-body dynamics theory. The vehicle-track coupling dynamic model is composed of two parts, the vehicle and the track, which are connected by wheel-track relationship. In this model, the wheel flat was considered as the change of wheel radius, and the displacement responses of different wheel flat lengths to the wheel-rail forces were simulated by establishing wheel profiles with different flat lengths.

### 3.1. The Vehicle Model

**Figure 5:**
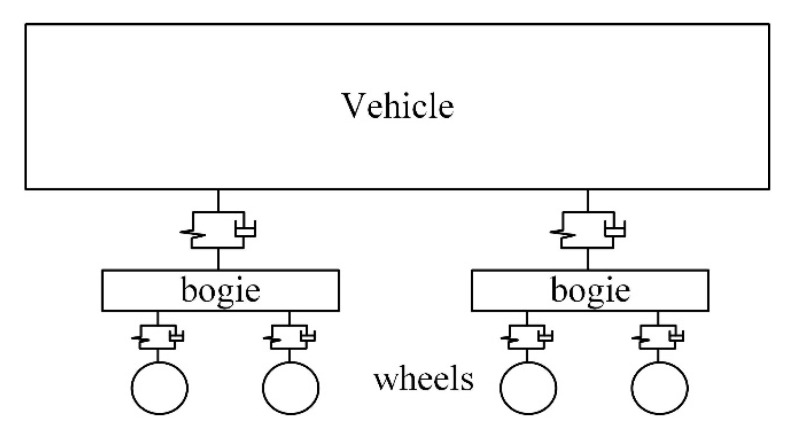
The schematic diagram of vehicle system dynamics model.

The dynamic model of vehicle system is shown in Figure 5. The multi-body system was composed of a car body, two bogies, four wheelsets, and a series of suspensions. The vehicle structure vibration equation can be expressed in the matrix form:

(6) $$M_{v}{\ddot{u}}_{v}+C_{v}{\dot{u}}_{v}+K_{v}u_{v}=Q_{v}$$

where $M_{v}$, $C_{v}$, and $K_{v}$ are the mass matrix, damping matrix, and stiffness matrix of a vehicle, respectively, which can be obtained based on the Lagrange equation; ${\ddot{u}}_{v}$, ${\dot{u}}_{v}$, and $u_{v}$ are acceleration, velocity, and displacement vectors of the vehicle, and $Q_{v}$ is the load vector of the vehicle. Parameters used in simulation were shown in Table 1.

### 3.2. The Track Model

**Figure 6:**
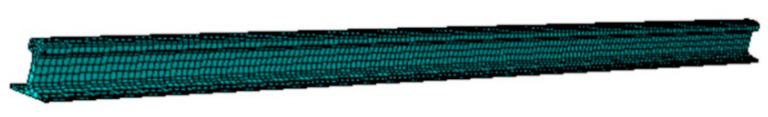
The finite element model of rail.

In order to simulate rail deformation, the flexible rail was used in the track model, so it is necessary to use the finite element structural analysis software ABAQUS CAE to deal with the rail flexibility. The solid model of the UIC-60 rails was imported into the finite element software for meshing, as shown in Figure 6. Then, the rail structure information file obtained from simulation was imported into the dynamics software SIMPACK for dynamics calculation. Based on the finite element theory, the motion equation of the element rail in the global coordinate system is:

(7) $$M_{R}{\ddot{u}}_{R}+C_{R}{\dot{u}}_{R}+K_{R}u_{R}=Q_{R}$$

where $M_{R}$, $C_{R}$, and $K_{R}$ are the mass matrix, damping matrix, and stiffness matrix of a rail, respectively, which can be obtained based on the Lagrange equation; ${\ddot{u}}_{R}$, ${\dot{u}}_{R}$ and $u_{R}$ are acceleration, velocity, and displacement vectors of the rail, and $Q_{R}$ is the load vector of the rail.

### 3.3. Mathematical Model of Wheel Flat

The newly appeared wheel flat can be equivalent to the string on the circumference of the wheel tread, but an ideal new wheel flat is not always present. When a new wheel flat appears, with the train running, the flat’s edge and corner of the wheel tread will be rounded quickly and become old flats. The test data in reference [24] shows that the wheel flats of the repaired vehicles are all old flats, which also shows that new wheel flat is rarely appeared. Therefore, it is necessary to establish a mathematical model which can describe the old wheel flat so as to simulate and analyze the impact of wheel flats. The established flat model is a cosine function related to flat length and vehicle running distance:

(8) $$\Delta Z\left(x\right)\;=\;\frac{1}{2}D_{f}[1\;-\;\cos(\frac{2\pi x}{L_{f}})]$$

where $D_{f}={L_{f}}^{2}/\left(16R\right)$, $L_{f}$ represents the wheel flat length, *R* represents the radius of the wheel rolling circle, and *x* represents the running distance of the vehicle. The relationship between wheel flat depth and wheel radius is as follows [24]:

(9) $$R(\theta )\;=\;R\;-\;\frac{Ra^{2}}{8}[1\;-\;\cos(\frac{\pi }{a}\theta )]$$

where $a\;=\;L_{f}/2R$, *θ* is the rotation angle of the wheel. The calculation results of this model can be loaded into the vehicle model to simulate the wheel-rail forces of different flat lengths.

## 4. Dynamic Performance Analysis

The vehicle-track coupling dynamic model was developed to simulate the rail vertical deformation caused by the wheel-rail interaction. The dynamic response of wheel flat to rail deformation was obtained by simulating different flat lengths with the model. According to the simulation results, the influencing factors of the measurement were analyzed and quantified.

### 4.1. Rail Bending Deformation

**Figure 7:**
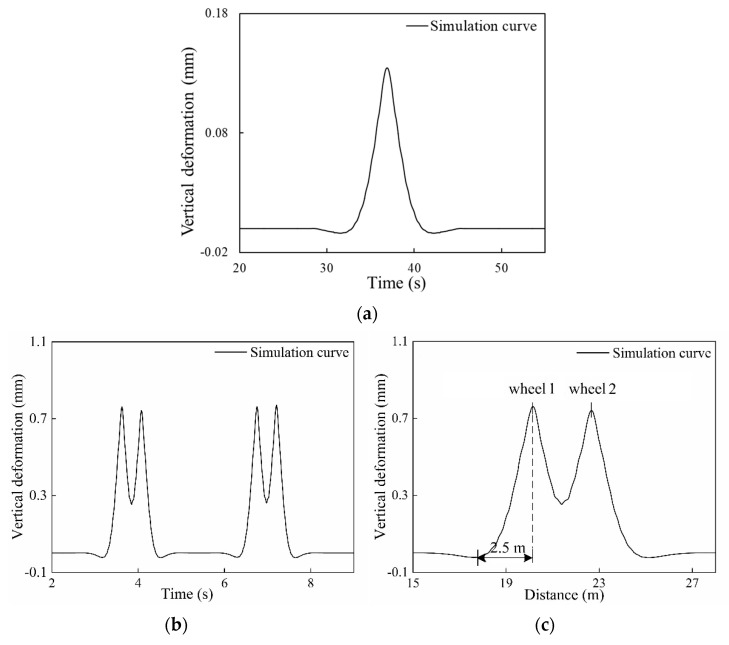
The response curve of the rail vertical displacement: (**a**) The vertical displacement response curve of the rail under the action of the wheel at 0.4 m/s; (**b**) The vertical displacement response curve of the rail under the action of the vehicle at 20 km/h; (**c**) The vertical displacement response curve of the rail under the action of the bogie at 20 km/h.

The wheelset model without load was developed to simulate the vertical displacement response of the rail. The vehicle speed was set to 0.4 m/s, as shown in Figure 7a. As the figure shows, the rail deformation first increases and then decreases under the wheel force. The vehicle-track coupling dynamic model was built to simulate the vertical displacement response of the rail as shown in Figure 7b,c. In Figure 7c, the rail is gradually deformed due to the influence of wheel 1 of the bogie, and the rail deformation displacement is the largest until the wheel reaches the position directly above the rail measurement point. After wheel 1 leaves, the rail deformation gradually decreases until wheel 2 begins to approach the measurement point and the rail deformation increases gradually. As shown in Figure 7c, it can be calculated that the length of the deformed area is in the range of ±2.5 m.

### 4.2. Influence of Wheel Flat on Rail Deformation

**Figure 8:**
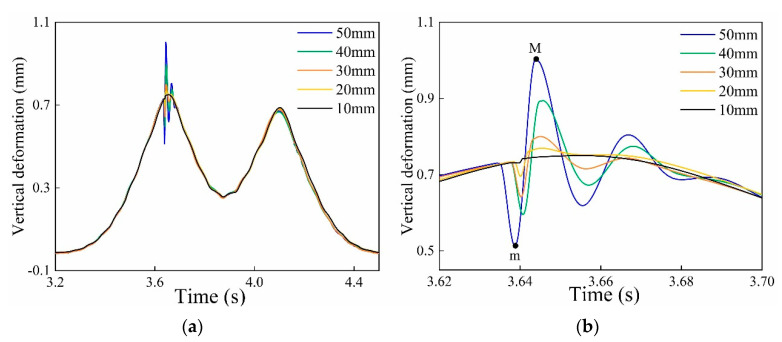
The impact response curves of the wheel flats at 20 km/h: (**a**) The impact response curves of the different flat lengths; (**b**) The flat signal waveforms.

The wheel flat mathematical model was established to analyze the influence of different flat lengths on rail deformation. Wheel profiles with different flat lengths (10 mm, 20 mm, 30 mm, 40 mm, and 50 mm) were simulated and the impact effect of different flat lengths on the same position of the rail was obtained, as shown in Figure 8a. An impact signal occurred in the rail deformation curve due to the vibration impact force of the wheel flat. In order to see the process clearly, the impact signals are enlarged and displayed in Figure 8b. As can be seen from the Figure 8b, the rail deformation decreases first and then increase. The minimum value point m and the maximum value point M appeared. The difference between the maximum value M and the minimum value m of rail vertical deformation was defined as the amplitude of the impact signal $M_{A}$, and it can be concluded that the amplitude of the flat signal $M_{A}$ increases with the increase in flat length.

### 4.3. Analysis of Influencing Factors

**Figure 9:**
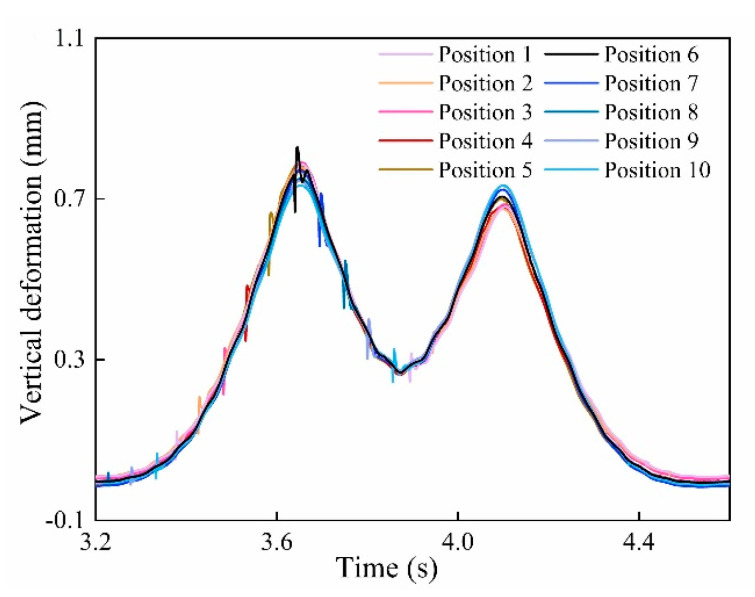
The impact response curves of the wheel flat under the different impact positions at 20 km/h.

The main factors affecting the wheel flat measurement are impact position, train speed, and load. The influence of these parameters was analyzed by the control variates method. A flat with 30 mm length was simulated on wheel 1 of the bogie, and the rail deformations under the different impact positions were analyzed. Each impact position was spaced 300 mm and the rail deformation curves under different impact positions were obtained and represented by different colors, as shown in Figure 9. As Figure 9 shows, the amplitude of the impact signals increases with the increase in the rail vertical deformation. Thus, it can be concluded that as the vertical deformation becomes larger, the flat detection is more sensitive. The maximum amplitude of the flat signal is represented by $M_{\max}$.

**Figure 10:**
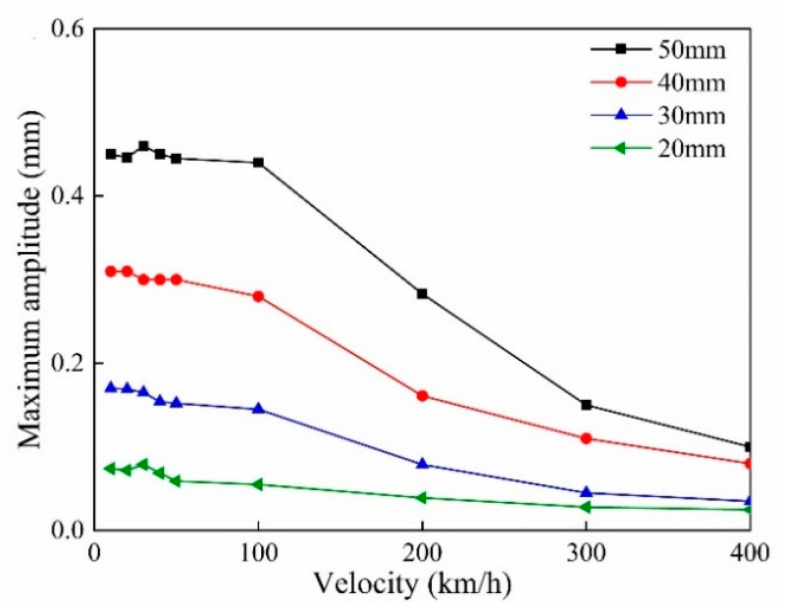
The impact response curves of the wheel flats at different speeds.

The wheel-rail contact force increases with the speed of the train, so it is necessary to analyze the dynamic response of the wheel flat to the wheel-rail system at different speeds. The relationship between train speed and the maximum amplitude $M_{\max}$ is shown in Figure 10, which demonstrates that as the vehicle speed increases, the maximum amplitude $M_{\max}$ of the flat signal decreases.

**Figure 11:**
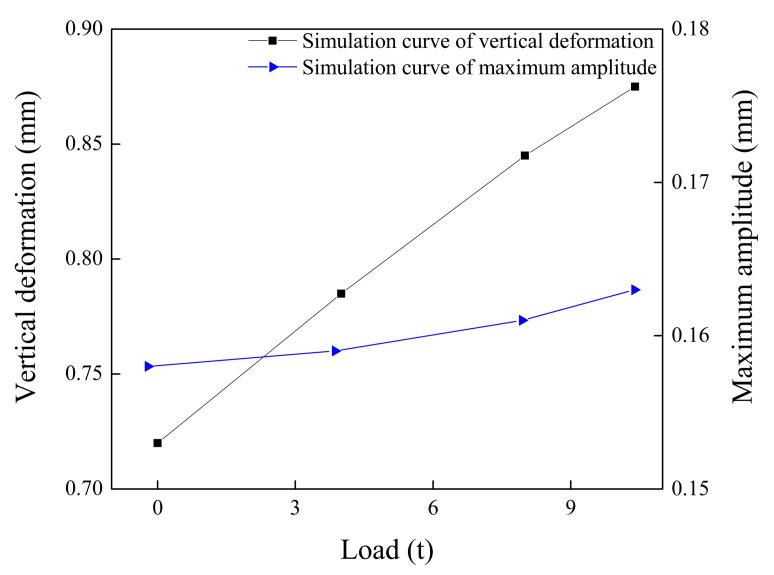
The impact response curves of the wheel flat under different loads.

Increasing the load of the train will also increase the wheel-rail contact forces. The relationship between the rail vertical deformation and the load is shown in Figure 11. As Figure 11 shows, the rail bending deformation will increase with the increase in the train load, but the load has less effect on the maximum amplitude.

### 4.4. The Quantification of Wheel Flat

**Figure 12:**
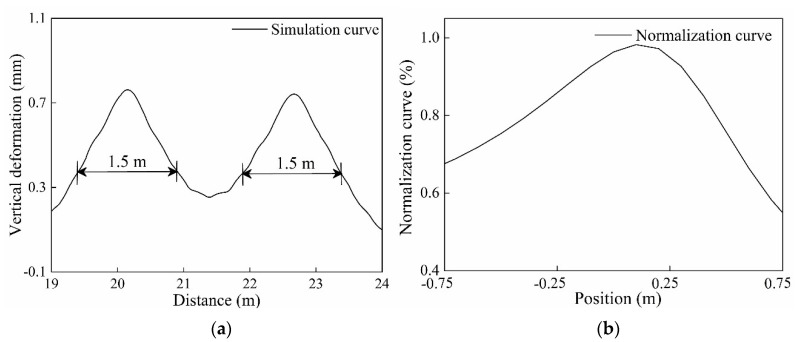
The detection area and the normalization curve: (**a**) The schematic diagram of the detection area; (**b**) The normalization curve of the wheel flat.

As described in Section 4.2, the amplitude of the flat signal increases with the increase in vertical deformation. In order to improve the sensitivity of wheel flat detection, the detection area was selected to be ±0.75 m from the maximum deformation position, as shown in Figure 12a. The simulation results are substituted into Equation (5) to obtain the output waveform of the detector. In the model, the distance between the laser and the cube-corner prism was set to 400 mm. The flat signal amplitude $M_{A}$ at different measuring positions in the detection area was obtained and normalized. The normalized curve was shown in Figure 12b with a fitting formula as follows:

(10) $$q\;=\;(0.392x^{2}\;-\;0.457x\;+\;0.417)/(x^{2}\;-\;0.608x+\;0.433)$$

where *x* is the flat position and *q* is the scale factor of the flat signal amplitude. The root-mean-square error of the fitting curve is 0.036 mm.

**Figure 13:**
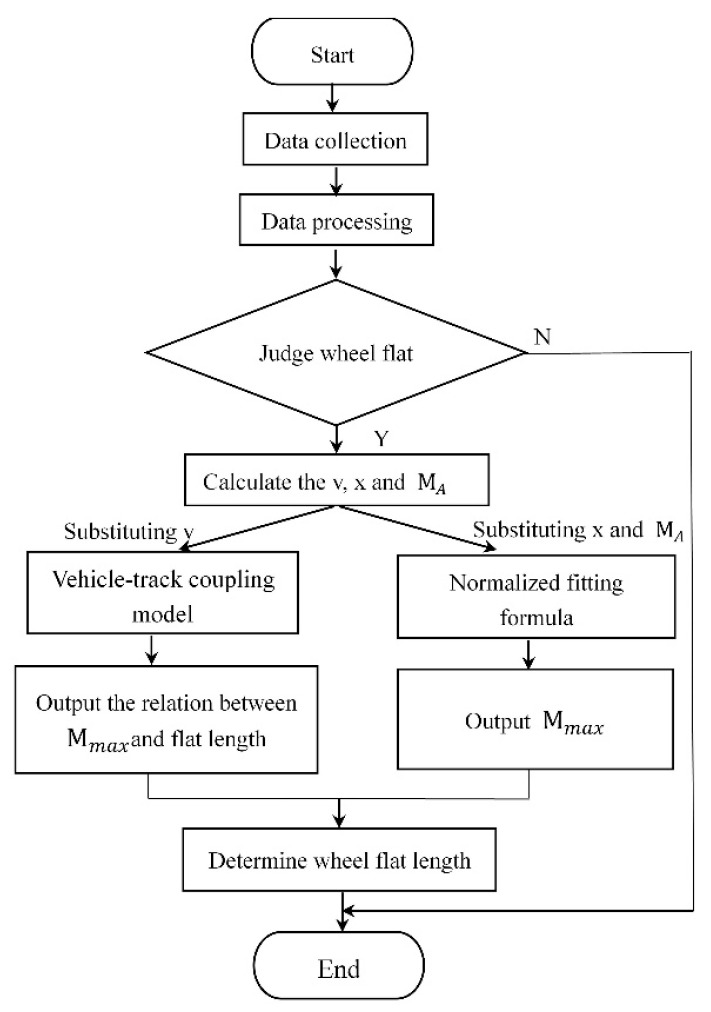
The flow chart of the wheel flat calculation.

**Figure 14:**
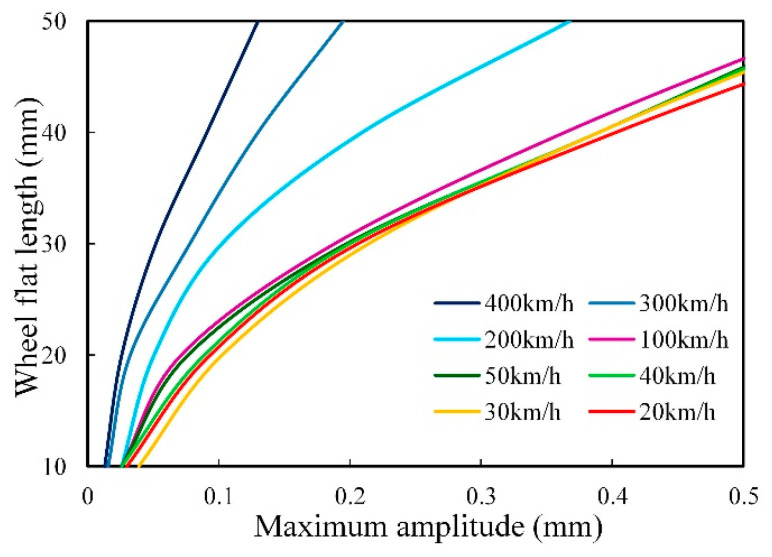
The relation curves between the maximum amplitude of the wheel flat and the flat length.

After the rail deformation curve with wheel flat was collected by the system, the calculation flow of quantifying flat length is shown in Figure 13. As Figure 13 shows, the impact signal amplitude $M_{A}$ and the flat position *x* can be obtained through data processing. The maximum amplitude of the flat signal $M_{max}\;=\;M_{A}/q$ can be calculated by substituting *x* into Equation (10). The vehicle speed can be calculated according to the wheelbase of bogie and the sampling frequency of the detector. Then, the speed is input into the vehicle-track coupling model and the rail displacement response curves of different flat lengths can be simulated. After data processing, the relationship curve between the maximum amplitude of the flat signal $M_{max}$ and the wheel flat length can be obtained. Part of the simulation results were shown in Figure 14. The actual wheel flat length can be further calculated according to the measured $M_{max}$ and the relation curve.

## 5. Results and Discussion

In the above sections, the measurement principle of the system was introduced, and the influencing factors of flats detection were analyzed by establishing the simulation model. The simulation results were normalized to realize quantitative measurement. In order to verify the effectiveness of the system, the laser collimation method-based wheel flat detection system was mounted in laboratories and railway sites, respectively, and experiments in the laboratory and on-site were conducted.

### 5.1. The Laboratory Experiment

**Figure 15:**
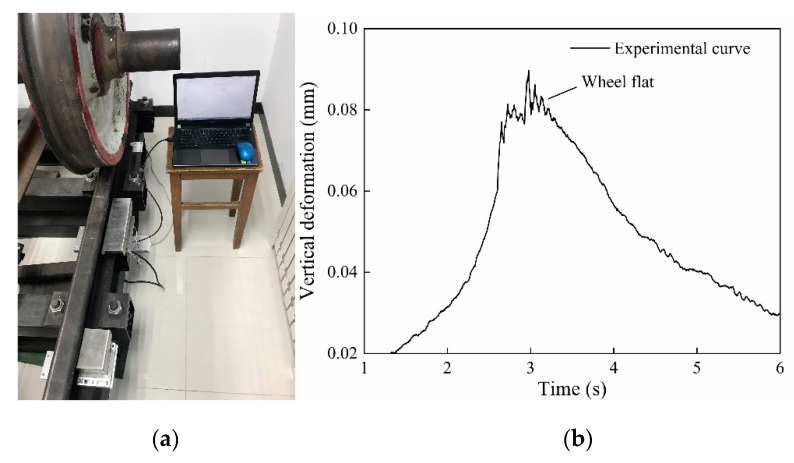
The wheel flat detection experiment: (**a**) The diagram of experimental devices; (**b**) The measurement waveform of wheel flat.

**Figure 16:**
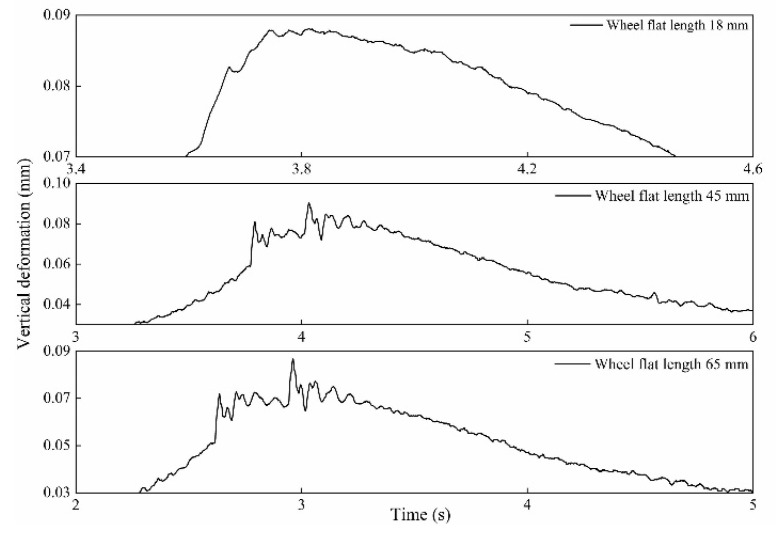
The measurement waveforms of three different wheel flat lengths.

**Figure 17:**
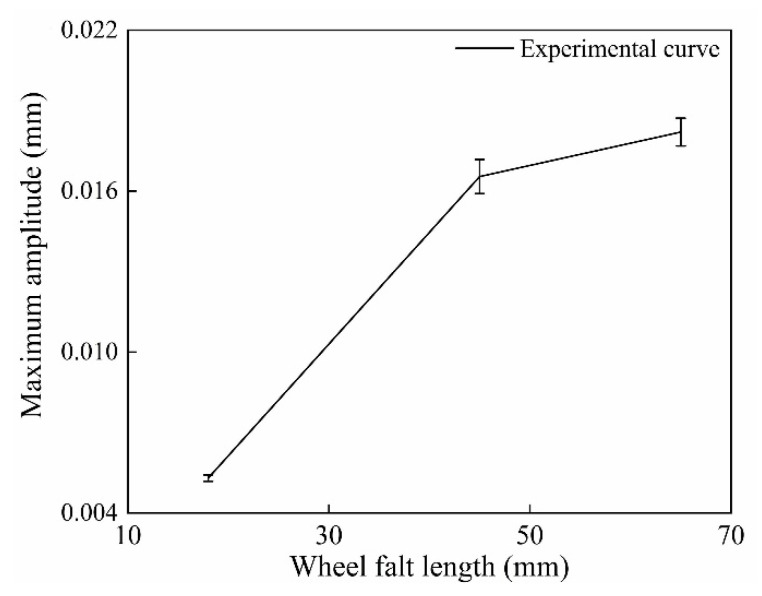
The error curve of the wheel flat.

In order to verify the simulation results, the sensor system was mounted in the laboratory for flat detection. In the experiment, different length and thickness of spacers were placed on the rail to simulate different wheel flat lengths. The experimental device was shown in Figure 15a. The wheelset without load was used in the experiment, and the obtained measurement curve after the wheel passed at 0.4 m/s was shown in Figure 15b, which is in good agreement with the transformation trend of the simulate waveform in Figure 7a. As Figure 15b shows, the impact signal in the rail vertical deformation curve reflects wheel flat. Three lengths of wheel flat were simulated in the experiment, and five repetitive experiments were performed for each length. The measurement waveforms of each flat length were shown in Figure 16. As Figure 16 shows, the flat signal amplitude increases with the increase in flat length, which is consistent with the simulation results. Data processing was performed to the measurement waveform, and the flat signal amplitude $M_{A}$ was extracted. The repeatability calculation results of $M_{A}$ are shown in Table 2 and the error curve is shown in Figure 17. It can be concluded that wheel flats of different lengths can be detected by the measurement system, and the smaller the flat length, the better the measurement repeatability.

### 5.2. The Field Test

**Figure 18:**
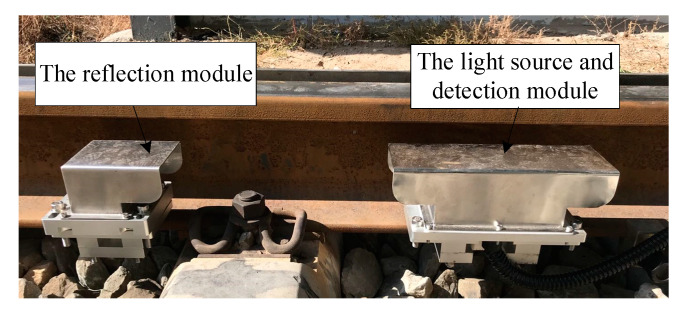
The field installation of the wheel flat detection system.

In order to verify the effectiveness of the system, field tests were performed in Yinchuan operation garage, China during October 2019. The system was installed in the garage entrance and the on-site installation of the sensor is shown in Figure 18.

**Figure 19:**
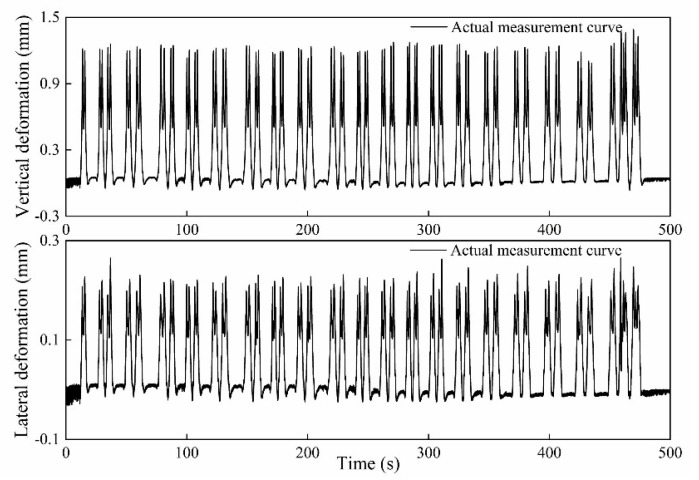
The vertical and lateral measuring waveforms on the site.

**Figure 20:**
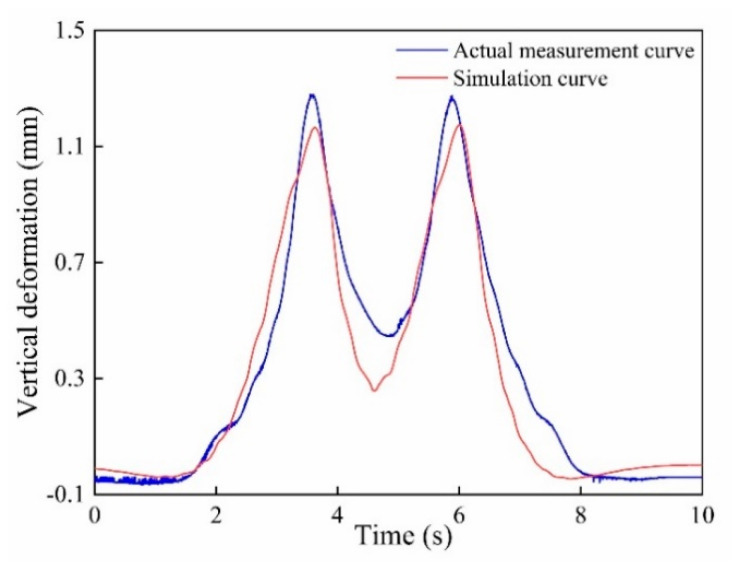
The simulation and experimental curves of the rail vertical deformation at 5 km/h.

The vertical and lateral measuring waveforms collected on site by the wheel tread flat detection system are shown in Figure 19 under a train speed of 5 km/h. The measured train without load was composed of 19 passenger carriages and 1 locomotive. As Figure 19 shows, the 82 peaks in the rail deformation waveform correspond to 76 wheelsets of 19 passenger carriages and 6 wheelsets of 1 locomotive, respectively. It can be concluded that the sensor system can completely detect the rail bending deformation of each wheel, which can verify the effectiveness of the measurement system. The vertical deformation curve of the measured train under no-load condition at 5 km/h was simulated and compared with the actual measurement curve as shown in Figure 20. It can be seen that the vehicle-track coupling dynamic model established in this paper can be used to simulate the dynamic response of rail deformation and displacement under the wheel-rail contact forces. The change of the rail vertical deformation in the simulation waveform is in good agreement with the transformation trend of the actual measurement waveform, which can verify the correctness of the model simulation results and the feasibility of the sensor system. No flat wheels were found during the measurement, which was consistent with the results of manual inspection.

## 6. Conclusions

In this paper, an on-site wheel flat detection system based on the self-developed reflective optical position sensor was proposed, which can realize the dynamic quantitative measurement of wheel flat. In order to quantify the relationship between the output signal and wheel flat length, the finite element method and the multi-body dynamics method were adopted to establish the vehicle-track coupling dynamics model. The wheel profiles with different flat lengths were simulated by the vehicle model, and the results were concluded. Based on the simulation results, flat length was quantified and the quantitative detection of wheel flat was realized. The sensor system was installed on the railway site for dynamic on-line detection of wheel flats. The obtained vertical deformation curve of the rails was in good agreement with the simulation results, which can prove the feasibility of this method. The effectiveness of the system has been verified at low speed. In future research, the experimental verification and quantitative detection of high-speed wheels will be performed.

## Figures and Tables

**Table 1:** Parameters used in simulation.

Parameters	Values	Parameters	Values
Mass of car body (kg)	4.72 × 10^4^	Pitch moment of inertia of bogie (kg·m^2^)	1205
Mass of bogie (kg)	3000	Yaw moment of inertia of bogie (kg·m^2^)	2792
Mass of wheel (kg)	1900	Vertical stiffness of primary suspension system [N/m]	8.865 × 10^5^
Roll moment of inertia of car body (kg·m^2^)	9.25 × 10^4^	Vertical stiffness of secondary suspension system [N/m]	2.03 × 10^5^
Pitch moment of inertia of car body (kg·m^2^)	1.756 × 10^5^	Vertical damping of primary suspension system[N·s/m]	6 × 10^3^
Taw moment of inertia of car body (kg·m^2^)	1.728 × 10^5^	Vertical damping of secondary suspension system[N·s/m]	8.4 × 10^4^
Roll moment of inertia of bogie (kg·m^2^)	1846		

**Table 2:** Wheel flat measurement results.

Wheel Flat Length (mm)	$M_{A}\;(\mathrm{mm})$				
L = 18	0.0052	0.0051	0.0052	0.0054	0.0054
L = 45	0.0157	0.0175	0.016	0.0168	0.0167
L = 65	0.0184	0.0182	0.0189	0.0173	0.0182
